# A Brainer on Neurotoxicity

**DOI:** 10.3389/ftox.2020.00003

**Published:** 2020-06-30

**Authors:** Ellen Fritsche, Helena Therese Hogberg

**Affiliations:** ^1^IUF-Leibniz Research Institute for Environmental Medicine, Düsseldorf, Germany; ^2^Medical Faculty, Heinrich-Heine-University, Düsseldorf, Germany; ^3^Center for Alternatives to Animal Testing (CAAT) at the Johns Hopkins Bloomberg School of Public Health, Baltimore, MD, United States

**Keywords:** neurotoxicity, DNT, human relevance, developmental neurotoxicity, susceptible life stages

We talk about adult neurotoxicity when exposure to natural or manmade toxic substances, interferes with normal nervous system activity and function. Neurotoxicity might disrupt or even kill neurons or the surrounding glia cells by interfering with neural function. Special attention must be given to susceptible subgroups such as, the very young (developmental neurotoxicity - DNT) and the aging population, as neurotoxicity's mode-of-action (MoA) or sensitivity differs for them vs. healthy adults. So far, neurotoxicity has been evaluated using animal models, rodent primary, or tumor *in vitro* cultures, and human epidemiology.

The field of neurotoxicology has benefitted tremendously from the field of drug development for nervous system disorders. High attrition rates, (at least 50%) in general (Smietana et al., 2016) and for central nervous system (CNS) drugs specifically (Arrowsmith and Miller, 2013; DiMasi, 2014; Harrison, 2016; Mohs and Greig, 2017), were attributed to insufficient human predictability of nonclinical biological data, i.e., efficacy and safety issues (Bailey et al., 2014; DiMasi, 2014; Harrison, 2016; Cavero et al., 2019). For CNS drug development, transgenic animals modeling human disease are widely used. The especially high failure rate of CNS drug developments is ascribed amongst others to the complexity of human CNS diseases that often involve multiple molecular targets. Furthermore, experimental animal disease models have relatively low predictive validity, and there is lack of established clinical biomarkers and proof-of-concept models for these diseases (Kola and Landis, 2004; Palmer and Stephenson, 2005). The failure rate in clinical trials is especially high for neurodegenerative disorders such as Alzheimer disease where 99.6% of drugs do not make it to the market (Pistollato et al., 2016; Mohs and Greig, 2017). Including human-relevant pharmacokinetics and bioavailability early into the drug development phase already reduced drug failure rates (Kola and Landis, 2004). Hence, improvement of the pharmaco-/toxicodynamics of models in health and disease for predicting not only drug effects but also the neurotoxicity of environmental chemicals, particulate matter, or other noxae will aid the development of better human prediction (Leist and Hartung, 2013; Cavero et al., 2019).

It was in the year 1993 when the use of stem cells for toxicological research was first proposed. It took 10 more years to first involve mouse embryonic stem cells in neurotoxicity evaluation. In 2006, human-induced pluripotent stem cells (hiPSC) entered the field and were first applied for neurotoxicity evaluation in 2016 (Barenys and Fritsche, 2018). For over three decades, almost, toxicity in general as well as neurotoxicity evaluation have seen dramatic changes. A future was envisioned in which toxicology relied primarily on high-throughput *in vitro* assays and computational models based on human biology to evaluate potential adverse effects of chemical exposures in a regulatory context (NRC, 2007). Moreover, the value of exposure science and epidemiology was strongly recognized for achieving human-relevant risk assessment in the future (NRC, 2012, 2017). In the area of neurotoxicity, these novel concepts have specifically and systematically been taken up for DNT. A global scientific network has been aimed at setting up a DNT *in vitro* testing battery consisting of primarily human stem/progenitor cell-based assays as well as zebrafish—as an alternative model organism—with the ultimate goal of application for regulatory purposes (Lein et al., 2005; Bal-Price et al., 2015, 2018; Fritsche et al., 2017, 2018; OECD, 2020). *In vitro* methodologies for studying acute neurotoxicity have also seen rapid advancement. Reproducible production of electrically active human neurons in cultures has been achieved, and the transition from 2D to 3D methods allows us to obtain complex models suitable for investigating neurotoxicity or brain-related diseases also with patient-derived cells (Grainger et al., 2018; Yla-Outinen et al., 2019). Thus, human iPSC-derived cells offer a platform with the unique advantage of almost unlimited availability and reproducing the “human context” *in vitro* by preserving the genetic and molecular phenotype of their donors. Despite this, stem cells differentiated into neurons and astroglia, which have been the most common cell types used for neurotoxicity testing so far, do not solely represent the wholeness of a brain. Novel strategies, for example, implementing oligodendrocytes (Pamies et al., 2017, 2018), microglia (Abreu et al., 2018), region-specific astrocytes (de Majo et al., 2020), and vasculature (Worsdorfer et al., 2020) in standard neurotoxicity test methods, need more attention. In addition, while brain organoids offer much promise, there is a need to improve both the reproducibility and throughput of these models so that they can reach their full potential for neurotoxicity and DNT testing (Sivitilli et al., 2020).

The Specialty Section Neurotoxicology in the journal *Frontiers in Toxicology* will focus on ‘Human Relevance’ as an overarching theme. Papers are encouraged to deal with cutting-edge research in *in vivo, in vitro, in silico*, and in epidemiological approaches for neurotoxicity evaluation of all life stages. Primary research as well as review articles of all areas touching on neurotoxicological research are welcome. The human relevance of these findings needs to be discussed irrespective of the methods applied. Articles will target a broad audience including academic and clinical researchers as well as toxicologist from industry and regulatory agencies. Thus, the Specialty Section Neurotoxicology of *Frontiers in Toxicology* aims at generating a community for neurotoxicologists worldwide.

## Author Contributions

EF and HH wrote the manuscript. All authors contributed to the article and approved the submitted version.

## Conflict of Interest

The authors declare that the research was conducted in the absence of any commercial or financial relationships that could be construed as a potential conflict of interest.
